# Management Strategies for Truncus Arteriosus: A Comparative Analysis of Staged vs. Primary Repair

**DOI:** 10.1007/s00246-025-03790-z

**Published:** 2025-01-30

**Authors:** Yasuyuki Kobayashi, Shunji Sano, Yuto Narumiya, Ayari Kimura, Etsuji Suzuki, Shingo Kasahara, Yasuhiro Kotani

**Affiliations:** 1 Department of Cardiovascular Surgery, Okayama University Graduate School of Medicine, Dentistry, and Pharmaceutical Sciences and Okayama University Hospital, 2-5-1 Shikatacho, Kitaku, Okayama, Japan; 2 Department of Pediatric Cardiac Surgery, Showa University Hospital Toyosu, Tokyo, Japan; 3 Department of Epidemiology, Okayama University Graduate School of Medicine, Dentistry, and Pharmaceutical Sciences, Okayama, Japan

**Keywords:** Truncus arteriosus, Staged repair, Primary repair, Pulmonary artery banding, Risk stratification

## Abstract

We reviewed the outcomes of truncus arteriosus repair (primary vs. staged repair incorporating bilateral pulmonary artery banding), focusing on survival, reintervention, and functional data. We analyzed 39 patients who underwent a first intervention for truncus arteriosus (staged, n = 19; primary, n = 20) between 1992 and 2022. The median follow-up period was 8.0 (2.2–13.2) years. Survival, freedom from reoperation, and freedom from catheter intervention were estimated using the Kaplan–Meier method. High-risk patients were defined as those with a weight ≤ 2.5 kg, ≥ moderate truncal valve regurgitation, interrupted aortic arch, or preoperative shock. In the staged group, patients with a median weight of 2.6 kg had a median intensive care unit stay of 5 days and no hospital mortality after bilateral pulmonary artery banding. At repair, the staged group had a larger conduit for the right ventricular outflow tract (14 vs. 12 mm; *P* = .008). Catheter intervention on the branch pulmonary artery was required in 67% of patients in the staged group, but right ventricular end-diastolic pressure at follow-up was comparable between the groups (*P* = .541). Survival rates were higher among high-risk patients in the staged group (87.5% vs. 21.4% at 15 years; *P* = .004) but were comparable between groups for standard-risk patients (*P* = 1.000). Bilateral pulmonary artery banding was a safe, effective procedure. Reintervention for branch pulmonary artery was common but did not affect functional outcomes. Staged repair may play a pivotal role regarding survival in high-risk patients, and risk stratification is vital.

**Supplementary Information:**

The online version contains supplementary material available at 10.1007/s00246-025-03790-z.

## Introduction

Truncus arteriosus, a complex congenital heart anomaly, typically requires surgical intervention during the neonatal period. Although primary neonatal repair is advocated, it is associated with high surgical mortality [1–3]. The Society of Thoracic Surgeons (STS) demonstrated that operative mortality was 10.8% even in recent years. Anatomic variations and patient conditions affect survival, with several identified as risk factors for mortality after repair [3–7]. Major adverse cardiac events (MACE) and postoperative complications remain common and have not significantly improved over time [4].

Bilateral pulmonary artery banding (bPAB), introduced in 1964 to temporarily stabilize patients with pulmonary overcirculation, is used for truncus arteriosus and ductal-dependent diseases [8]. Despite its decline due to concerns about immediate desaturation and long-term pulmonary artery stenosis, we adopted staged repair incorporating bPAB in 2006. Early outcomes of bPAB have been reported from the Japanese Cardiovascular Surgery Database, but late outcomes have not [9]. This study reviewed the early and late surgical outcomes, including mortality, reintervention (surgical or catheter), and functional data after definitive repair (primary or staged) for truncus arteriosus over 20 years.

## Patients and Methods

### Ethical Statement

This study was approved by the hospital’s institutional review board (approval number: 2009–023; approval date: October 23, 2020). The requirement for written informed consent was waived due to the study’s retrospective nature.

This single-institution retrospective review included 39 patients who underwent their initial intervention for truncus arteriosus (staged repair, n = 19; primary repair, n = 20) at Okayama University Hospital between September 1992 and August 2022. Preoperative, intraoperative, postoperative, and follow-up data were extracted from patient medical records and compared between the two groups.

### Operative Technique

Surgical intervention was indicated for symptomatic neonates and young infants with congestive heart failure. Primary repair was exclusively performed until 2005, but in 2006, bPAB as staged repair was introduced to all patients except those presenting late. Since 2017, staged repair was limited to patients with a weight ≤ 2.5 kg, interrupted aortic arch (IAA), preoperative shock, or other organ dysfunction. These patients were enrolled on the waiting list when they weighed > 6.0 kg. Management decisions were made in a multidisciplinary meeting.

Band tightness at bPAB was determined by body weight and adjusted using specific sutures or expanded polytetrafluoroethylene (ePTFE) conduits as follows: weight < 2.0 kg, 8-mm CV-2 suture (W.L. Gore and Associates, Flagstaff, AZ, USA); weight 2.0–2.5 kg, 3-mm ePTFE conduit; weight 2.5–3.0 kg, 3.5-mm ePTFE conduit; weight 3.0–3.5 kg, 4-mm ePTFE conduit with a width of 2 mm. Criteria for successful banding included a rise in systemic blood pressure ≥ 60 mmHg and maintaining adequate arterial oxygen saturation (mid-80 s%) and partial pressure of oxygen (PaO2; around 50 mmHg) with a fraction of inspired oxygen of 0.21.

### Study Endpoints, Measurements, and Definition

The primary endpoints were mortality, reintervention (surgical or catheter), and long-term functional data after definitive repair. Secondary endpoints included outcomes following bPAB. Two-dimensional, M-mode, and Doppler images were repeatedly obtained by cardiologists specializing in echocardiography in conformance with the American Society of Echocardiography guidelines. Truncal valve Z-scores were calculated based on the aortic valve [10].

Patients undergoing bPAB had a heart catheterization before definitive repair to assess branch pulmonary artery (PA), PA index (Nakata index), the pulmonary to systemic blood flow ratio (Qp/Qs), and pulmonary vascular resistance.

Mechanical ventilatory support was defined as the preoperative need for intubation. Preoperative shock was defined as a pH < 7.2 or a lactate level > 4 mg/dL [11]. High risk for early mortality after definitive repair was defined as weight ≤ 2.5 kg, ≥ moderate truncal valve regurgitation, IAA, or preoperative shock, based on published preoperative risk factors (Online resource 1) [3–7]. Other patients were considered standard risk.

### Follow-up

Follow-up was completed in September 2023. The median follow-up duration from the first intervention was 8.0 (2.2–13.2) years (staged group: 8.5 [4.9–11.8] years; primary group: 5.2 [0.5–23.4] years). In the year prior to the study’s conclusion, 37/39 (95%) hospital survivors underwent complete follow-up with echocardiography. Elective follow-up catheters were scheduled one year after definitive repair, at preschool age, and before adulthood.

### Statistical Analysis

Continuous variables were compared using the *t-*test or Mann–Whitney U test and reported as the mean ± SD or the median (interquartile range). Categorical variables were compared using Pearson’s χ^2^ test or Fisher’s exact test (if expected frequency was < 5) and reported as the absolute frequency (percentage). Survival, freedom from reoperation, and freedom from catheter intervention were estimated using the Kaplan–Meier method, with patients without these events censored at the end of follow-up. Truncal valve regurgitation grades before bPAB and definitive repair were compared using the Wilcoxon matched-pairs signed-rank test. *P*-values < 0.05 (two-sided) were considered statistically significant. Analyses were performed using Stata v.17 (StataCorp, College Station, Texas, USA) and GraphPad Prism software v.9 (San Diego, CA, USA).

## Results

### Demographics

Baseline characteristics at the first intervention are summarized in Table 1. The median age and weight for the cohort were 18 (13–34) days and 2.7 (2.5–3.2) kg, respectively. The staged group had a smaller median weight than the primary group (2.6 vs. 3.1 kg; *P* < 0.001). A higher percentage of staged patients had their first intervention between 2006 and 2022 (95% vs. 30%; *P* < 0.001).

**Table 1:** Patient characteristics at the first intervention

Characteristics	Staged group	Primary group	*P*-value
	(n = 19)	(n = 20)	
Male sex	8 (42)	11 (55)	0.421
Age (days)	15 (9–24)	21 (13–47)	0.143
Neonates	16 (84)	13 (65)	0.273
Weight (kg)	2.6 (2.2–2.7)	3.1 (2.7–3.5)	< 0.001
Weight ≤ 2.5 kg	9 (47)	3 (15)	0.041
Prematurity	2 (11)	0 (0)	0.231
DiGeorge syndrome	1 (5)	1 (5)	1.000
Van Praagh classification			0.333
Type A1	10 (53)	11 (55)	
Type A2	5 (26)	8 (40)	
Type A3	0 (0)	0 (0)	
Type A4	4 (21)	1 (5)	
Truncal valve morphology			0.044
Bicuspid	1 (5)	1 (5)	
Tricuspid	17 (89)	12 (60)	
Quadricuspid	1 (5)	7 (35)	
≥ Moderate truncal valve regurgitation	1 (5)	3 (15)	0.601
≥ Moderate truncal valve stenosis	0 (0)	1 (5)	1.000
Coronary artery abnormality	2 (11)	0 (0)	0.231
Extracardiac abnormality	4 (21)	2 (10)	0.401
Mechanical ventilatory support	4 (21)	7 (35)	0.480
Shock	2 (11)	2 (10)	1.000
Year of intervention			< 0.001
1991–2005	1 (5)	14 (70)	
2006–2022	18 (95)	6 (30)	
Risk stratification			0.341
Standard risk	9 (47)	13 (65)	
High risk	10 (53)	7 (35)	

Data are presented as the median (interquartile range) or n (%)

### Outcomes of bPAB

Outcomes of bPAB are described in Table 2. The band material and size varied, with 84% using 3.0- or 3.5-mm ePTFE conduits. Postoperatively, arterial oxygen saturation levels and PaO2 were 87% (85–89%) and 49 (43–50) mmHg, respectively. The median intensive care unit (ICU) stay was 5 (3–7) days, with no hospital mortality. One patient (2.6 kg), who underwent bPAB with a 3.0-mm ePTFE conduit and had PaO2 of 42 mmHg at the end of bPAB, required band replacement with a 3.5-mm ePTFE conduit on postoperative day 5. One patient (Van Praagh Type 2) was lost to follow-up after discharge. All four patients with IAA discontinued prostaglandin and were discharged without a ductal stent. The median duration from bPAB to definitive repair was 382 (247–557) days.

**Table 2:** Outcomes of bilateral pulmonary artery banding

Characteristics	Staged group (n = 19)	
Intraoperative		
Band material		
CV-2 suture	2 (11)	
3.0-mm ePTFE conduit	7 (37)	
3.5-mm ePTFE conduit	9 (47)	
4.0-mm ePTFE conduit	1 (5)	
Systolic blood pressure at the end of the procedure (mmHg)	70 (62–80)	
Diastolic blood pressure at the end of the procedure (mmHg)	37 (22–45)	
Arterial oxygen saturation level at the end of the procedure (%)	87 (85–89)	
Partial pressure of oxygen at the end of the procedure (mmHg)	49 (43–50)	
Concomitant procedure	0 (0)	
Postoperative		
Length of intubation (days)	2 (1–2)	
Length of stay in the intensive care unit (days)	5 (3–7)	
Hospital mortality	0 (0)	
Additional procedure during interstage	1 (5)	
Interval to definitive repair (days)	382 (247–557)	

Echocardiography	Before bPAB	Before repair	*P*-value
≥ Moderate truncal valve regurgitation	1 (5)	0 (0)	0.487
Truncal valve Z-score	6.2 ± 1.5	6.3 ± 1.6	0.856
Peak flow velocity across the band (m/s)			
Right	–	4.0 (3.7–4.2)	–
Left	–	4.0 (3.9–4.2)	–
Heart catheterization before definitive repair			
Pulmonary vascular resistance (Wood units)	1.8 (1.4–2.0)		
Qp/Qs	0.62 (0.53–0.90)		
Pulmonary artery index (Nakata index)	389 (240–420)		
Brain-type natriuretic peptide before definitive repair (pg/mL)		45 (23–62)	

Data are presented as the median (interquartile range), n (%), or mean ± SD

*ePTFE* expanded polytetrafluoroethylene, *Qp/Qs*, pulmonary to systemic blood flow ratio

Pre-bPAB and pre-repair ≥ moderate truncal valve regurgitation and Z-scores were comparable (*P* = 0.487 and 0.856, respectively). Pulmonary vascular resistance before repair was 1.8 (1.4–2.0) Wood units.

### Outcomes of Definitive Repair

Outcomes of definitive repair are shown in Table 3. The median age and weight at definitive repair in the staged group were 400 (267–567) days and 7.2 (6.0–8.7) kg, respectively. Branch PA patch plasty at the band site was performed in eight patients (44%) in the staged group, five of whose band material was either 3.0-mm ePTFE conduit or CV-2. Other patients only had dilatation with a Hegar dilator that was the same size as the normal size of the branch PA. The staged group had a larger median right ventricle (RV)-PA conduits (14 vs. 12 mm; *P* = 0.008), a smaller RV-PA conduit size/body surface area (39 vs. 58 mm/m^2^; *P* = 0.004), less frequent delayed sternum closure (11% vs. 55%; *P* = 0.006), and shorter intubation durations (2 vs. 5 days; *P* = 0.038).

**Table 3:** Outcomes of definitive repair

Characteristics	Staged group (n = 18)	Primary group (n = 20)	*P*-value
Age (days)	400 (267–567)	21 (13–47)	< 0.001
Neonates	0 (0)	13 (65)	< 0.001
Weight (kg)	7.2 (6.0–8.7)	3.1 (2.7–3.5)	< 0.001
Weight ≤ 2.5 kg	0 (0)	3 (15)	0.232
Echocardiographic data			
Truncal valve regurgitation ≥ moderate	0 (0)	3 (15)	0.232
Shock	0 (0)	2 (10)	0.488
Intraoperative results			
Aortic cross-clamp time (min)	141 ± 49	103 ± 18	0.003
Cardiopulmonary bypass time (min)	198 ± 60	184 ± 49	0.456
Concomitant procedure			
Arch repair	3 (17)	1 (5)	0.328
Branch pulmonary artery patch plasty	8 (44)	–	–
Truncal valve repair	2 (11)	4 (20)	0.663
RV-PA conduit size (mm)	14 (14–16)	12 (12–14)	0.008
RV-PA conduit size/body surface area (mm/m^2^)	39 (36–45)	58 (50–61)	0.004
Intraoperative RV pressure/LV pressure	0.56 (0.46–0.65)	0.53 (0.47–0.63)	0.915
Delayed sternum closure	2 (11)	11 (55)	0.006
Postoperative results			
Complications			
Extracorporeal membrane oxygenation	0 (0)	2 (10)	0.488
Mediastinal exploration	2 (11)	2 (10)	1.000
Length of intubation	2 (2–6)	5 (4–8)	0.038
Length of stay in intensive care unit	6 (4–11)	8 (7–14)	0.058
Hospital mortality	0 (0)	4 (20)	0.107
Late mortality	1 (6)	1 (5)	1.000
Surgical reintervention			
Interval to reintervention (years)	7.5 ± 4.4	3.9 ± 2.8	0.076
on RV-PA connection	8 (44)	7 (35)	0.741
on branch PA	7 (39)	4 (20)	0.288
Catheter reintervention			
Interval to reintervention (years)	5.5 ± 4.7	6.8 ± 7.7	0.646
Pulmonary artery balloon dilatation	12 (67)	8 (40)	0.119
Distal anastomosis site	6 (33)	8 (40)	0.745
Banding site	6 (33)	–	–
Long-term follow-up echocardiography			
≥ Moderate truncal valve regurgitation	2 (11)	4 (20)	0.663
Follow-up heart catheterization			
RV pressure/LV pressure	0.60 (0.44–0.69)	0.59 (0.53–0.80)	0.429
RV end-diastolic pressure (mmHg)	6 (5–8)	7 (5–9)	0.541
PA index (Nakata index)	284 (247–396)	407 (216–560)	0.898
Brain-type natriuretic peptide at latest follow up (pg/mL)	46 (24–89)	49 (27–110)	0.639

Data are presented as the median (interquartile range), n (%), or mean ± SD

*LV* left ventricle, *PA,* pulmonary artery, *RV* right ventricle

No hospital mortality occurred in the staged group, while four deaths occurred in the primary group (*P* = 0.107). Late mortality occurred in one patient in each group. Demographic and course details are summarized in Online Resource 2.

**Fig. 1:**
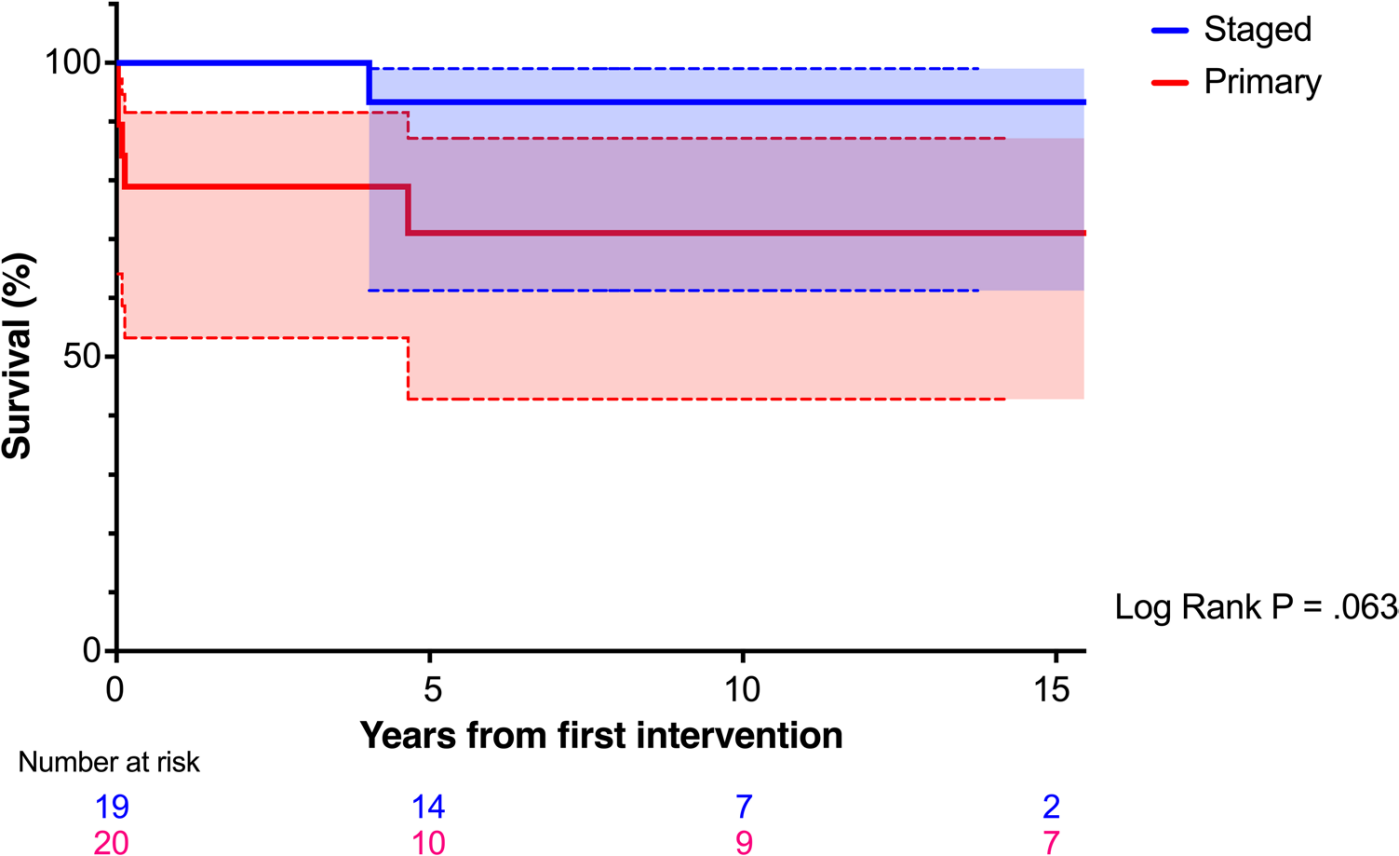
Kaplan–Meier curves for survival, stratified by surgical strategy. Color shading represents the 95% confidence intervals

The survival probabilities (95% confidence interval [CI]) at 5, 10, and 15 years were 93.8% (63.2–99.1%), 93.8% (63.2–99.1%), and 93.8% (63.2–99.1%) in the staged group and 71.8% (44.3–87.4%), 71.8% (44.3–87.4%), and 71.8% (44.3–87.4%) in the primary group, respectively (*P* = 0.063; Fig. 1).

**Fig. 2:**
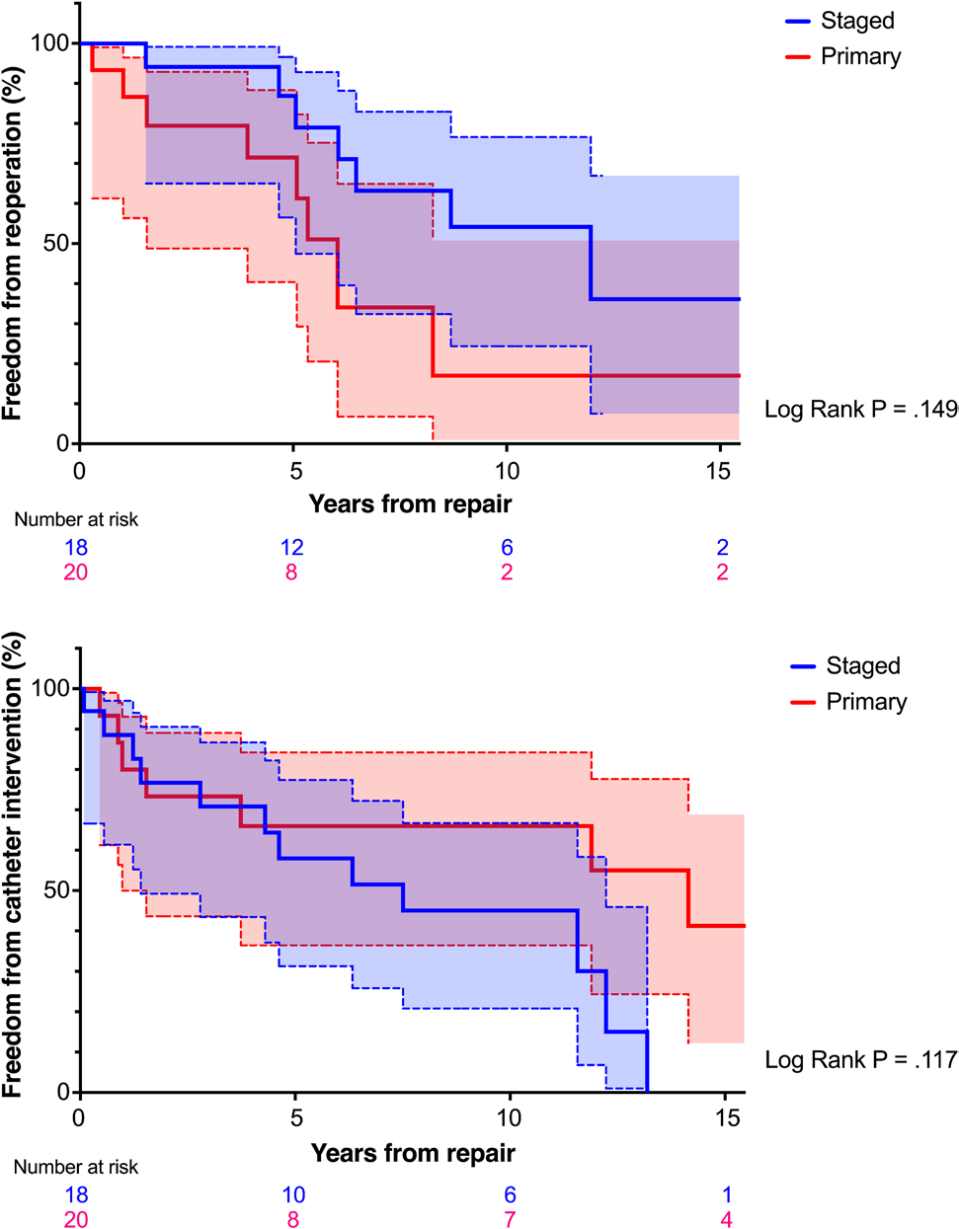
Kaplan–Meier curves for functional outcomes, stratified by surgical strategy. **a** Freedom from reoperation. **b** Freedom from catheter intervention. Color shading represents the 95% confidence intervals

RV-PA connection or branch PA reoperations occurred in 16 (42%) patients (staged: 8/18; primary: 8/20). Freedom from reoperation (95% CI) at 5, 10, and 15 years was 86.9% (56.5–96.6%), 54.2% (24.4–76.6%), and 36.1% (7.5–67.0%) in the staged group and 71.5% (40.4–88.3%), 17.0% (1.0–50.8%), and 17.0% (1.0–50.8%) in the primary group (*P* = 0.149; Fig. 2a). Cather-based balloon dilatation was required in 23 (53%) patients (staged: 12/18; primary: 8/20), with six staged patients requiring intervention on previous banding sites. Freedom from catheter intervention (95% CI) at 5, 10, and 15 years was 58.0% (31.3–77.4%), 45.1% (20.8–66.7%), and 0% in the staged group and 66.0% (36.5–84.3%), 66.0% (36.5–84.3%), and 41.3% (12.3–68.8%) in the primary group (*P* = 0.117; Fig. 2b).

Follow-up heart catheterization demonstrated comparable RV pressure/LV pressure and RV end-diastolic pressure between groups (*P* = 0.429 and 0.541, respectively). B-type natriuretic peptide levels were also comparable (*P* = 0.639).

### Survival Comparison Stratified by Surgical Risk and Strategy

**Fig. 3:**
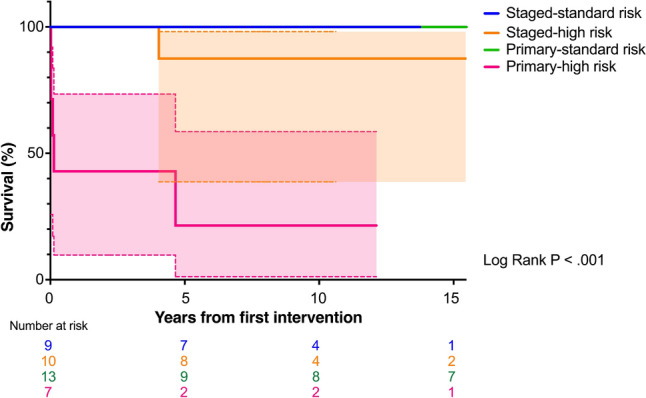
Kaplan–Meier curves for survival, stratified by surgical strategy and risk. Color shading represents the 95% confidence intervals

The cohort was divided by surgical risk (standard or high risk) and strategy (staged or primary repair). No mortality occurred in standard-risk patients, regardless of strategy. The survival probabilities (95% CI) at 5, 10, and 15 years were 100% in both the staged risk and primary standard-risk patients, 87.5% (38.7–98.1%) in staged high-risk patients, and 21.4% (1.2–58.6%) in primary high-risk patients (*P* < 0.001; Fig. 3). Survival was comparable among standard-risk patients (*P* = 1.000) but higher among high-risk staged patients (*P* = 0.004).

## Discussion

**Fig. 4:**
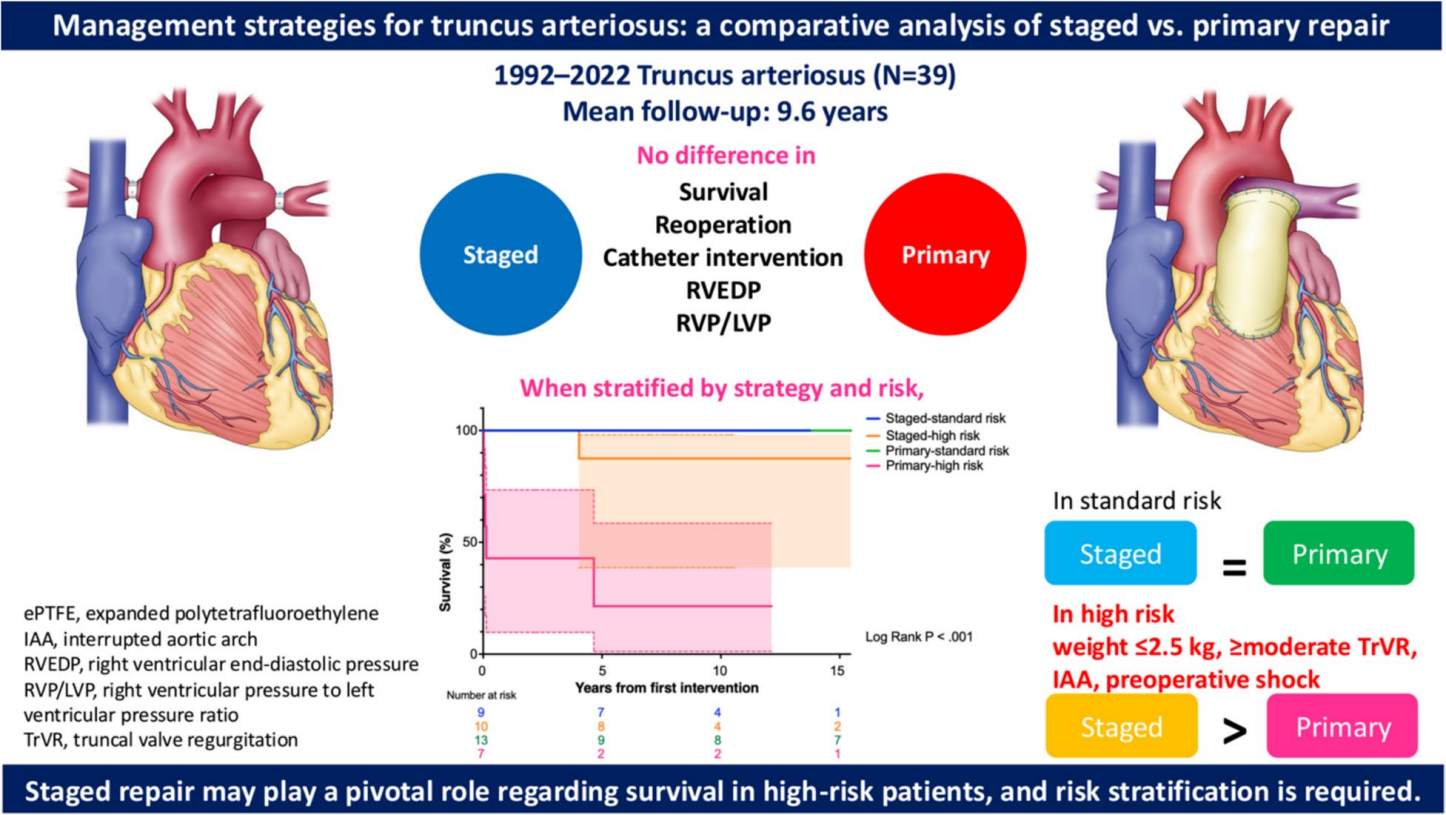
Surgical approaches for truncus arteriosus. Staged repair may be a good alternative to primary repair for high-risk patients

This study highlights the safety and effectiveness of bPAB, with key findings including the short length of intubation and ICU stays, infrequent reinterventions, no complications or hospital mortality after bPAB, low pulmonary vascular resistance before definitive repair, and less delayed sternal closure and no mortality after definitive repair. The staged repair group had comparable reoperation and RV function to the primary repair group, although catheter reinterventions for the banding sites were not uncommon. Moreover, survival probabilities were comparable between staged and primary repairs for standard-risk patients but higher for high-risk patients in the staged group. Therefore, risk stratification is essential for improving staged repair outcomes in high-risk cases (Fig. 4).

### High Risk for Primary Repair

Truncus arteriosus repair, which involves ventricular septal defect closure and RV outflow tract reconstruction, has remarkably high mortality and complication rates [3, 4]. Primary repair during the neonatal period jeopardizes RV function due to a large ventriculotomy to accommodate the conduit and high pulmonary vascular resistance, particularly when performed within the first week, often requiring nitric oxide and an open sternum. Therefore, mortality and complications may increase when repair is performed in high-risk patients.

The definition of high risk for morality after repair varies slightly among publications but is broadly consistent. Previous studies have identified low weight at repair (threshold of 2.5 kg), truncal valve regurgitation or the need for truncal valve repair, IAA, and shock. However, prematurity, coronary anomalies, and DiGeorge syndrome have not been identified as high-risk factors (Table 1) [3–7]. Low weight poses a technical challenge due to the small heart size, potentially resulting in a larger conduit, which is an intraoperative risk factor for hospital mortality [4, 7]. Despite surgical advances, truncal valve regurgitation remains a risk [12, 13]. Goyal et al. described that intervening in moderate truncal valve disease at repair may not improve outcomes, as truncal valve regurgitation often diminishes after volume load reduction, and there is no consensus to intervene in moderate truncal valve regurgitation, although severe regurgitation should be addressed [3]. IAA is recognized as the highest risk in the STS – European Association for Cardio-Thoracic Surgery Congenital Heart Surgery Mortality Categories [14]. Preoperative shock, though rarely investigated, increases the risk of MACE in the immediate postoperative period [4]. Patients with these preoperative factors in this study benefited from staged repair.

Overall, high-risk patients avoided mortality through staged repair, while normal-risk patients underwent primary definitive repair, avoiding DSC and achieving short intubation periods and ICU stays. By avoiding primary repair in high-risk patients, we minimized the risk of prolonged intubation and ICU stays and subsequent complications associated with more complex recoveries.

### Benefits and Burdens of Staged Repair

bPAB, a palliative procedure to regulate excessive pulmonary blood flow, offers several benefits. First, bPAB skips extensive cardiac reconstruction, cardiopulmonary bypass, and RV failure during the neonatal period, reducing the likelihood of an open sternum or postoperative extracorporeal membrane oxygenation. The short intubation time and ICU stay without mortality underscore its safety. Second, by controlling pulmonary overcirculation, bPAB effectively manages congestive heart failure. No cases required conversion to definitive repair post-bPAB, and brain-type natriuretic peptide levels were near normal before repair. Pre-repair heart catheterization demonstrated favorable lung conditions (i.e., low pulmonary vascular resistance) with regulated Qp/Qs and a preserved PA index. Third, deferred repair permits the use of a larger RV-PA conduit, potentially extending the time before another conduit change and reducing lifetime reoperations. Avoidance of large RV-PA conduits/body surface areas associated with MACE may contribute to infrequent delayed sternum closure in the staged group [4].

Successful bPAB relies on a rigid protocol for band material and size based on patient weight, along with careful decision-making during the procedure, similar to the main PA banding procedure [15, 16]. Although precise band adjustment is rarely needed, measuring oxygen saturation and PaO_2_ at the end of bPAB is indispensable to ensuring adequate pulmonary blood flow. The target peak velocity across each band is 3.5 m/s, but this is not always the decision-making tool due to occasionally elevated pulmonary vascular resistance. This is why we avoided bPAB in the first week, with only 1/19 patients undergoing it in that period.

bPAB generally has two concerns: physiology after bPAB and branch PA stenosis. In truncus arteriosus, bPAB increases antegrade blood flow to the brain, lower body, and coronary arteries, differing from conditions like hypoplastic left heart syndrome, in which flow sometimes depends on retrograde flow from the isthmus (Table 3) [17, 18]. Even patients with IAA discharged without prostaglandin or a ductal stent experienced no complications during the interstage period. Branch PA stenosis after bPAB is a significant concern [19, 20]. About half of the patients in our cohort required patch plasty for the banded PA at definitive repair, and freedom from catheter intervention at 15 years was 0%. However, no patients required multiple catheter or surgical interventions for banded PA sites, and RV pressure/LV pressure and RV end-diastolic pressure were comparable between groups. This suggests that branch PA stenosis after bPAB did not significantly impact functional outcomes over the 15-year observation period, although each patient required at least one PA-related reintervention. Further follow-up is required to determine whether branch PA stenosis poses a long-term burden.

### Staged Repair for High-risk Patients

Although bPAB is not recommended for every patient due to potential burdens and comparable survival probabilities among standard-risk patients, staged repair incorporating bPAB may benefit high-risk patients due to its higher survival probability. Attention is required for truncal valve regurgitation, as volume reduction alone may not improve regurgitation primarily caused by dysplastic leaflets. Therefore, identifying suitable candidates based on truncal regurgitation etiology is crucial. High-risk definitions vary by institution, and we are not advocating a uniform definition. The surgical approach should be individualized based on the definition and presence of high-risk features, which is where a staged strategy may play a pivotal role regarding survival.

## Limitations

This study had several limitations. Echocardiographic data, performed by a few specialized cardiologists, were not reviewed by core labs or examiners, potentially introducing reader variability. Although 95% of hospital survivors completed follow-up within 1 year, the median follow-up was 8.0 years, representing a significant gap. The small sample size included only one patient with ≥ moderate truncal valve regurgitation in the staged group, making it difficult to assess the effectiveness of staged repair for this subgroup, despite bPAB not worsening truncal valve regurgitation. Defining time zero for reoperation rates as the point of definitive repair, rather than the initial staged procedure, is a methodological choice that may lead to controversy. While this approach aligns with recent studies, it could introduce potential bias by excluding the initial intervention as part of the analysis. This limitation should be considered when interpreting our findings. Additionally, being a single-institution study, the generizability of our staged approach may be limited.

## Conclusions

bPAB was a safe and effective procedure that enabled the use of a larger conduit at definitive repair. Reintervention for branch PA was not uncommon but did not affect functional outcomes, although further follow-up is required. Staged repair may be a good alternative to primary repair for high-risk patients, and risk stratification is vital.

## Supplementary Information

Below is the link to the electronic supplementary material.

Supplementary file1 (DOCX 59 KB)

Supplementary file2 (DOCX 57 KB)

## Data Availability

No datasets were generated or analysed during the current study.
