# Supplementary material for: Management Strategies for Truncus Arteriosus: A Comparative Analysis of Staged vs. Primary Repair
Source: Pediatr Cardiol. 2025 Jan 30;47(2):514–22. doi: 10.1007/s00246-025-03790-z (PMC12855218; doi:10.1007/s00246-025-03790-z)
Supplement: Supplementary file 1 — Supplementary file1 (DOCX 59 KB) [file 246_2025_3790_MOESM1_ESM.docx]

**Management strategies for truncus arteriosus: a comparative analysis of staged vs. primary repair**

*Pediatric Cardiology*

Yasuyuki Kobayashi, Shunji Sano, Yuto Narumiya, Ayari Kimura, Etsuji Suzuki, Shingo Kasahara, Yasuhiro Kotani*

*Corresponding Author:

Yasuhiro Kotani, MD, PhD

Department of Cardiovascular Surgery

Okayama University Graduate School of Medicine, Dentistry, and Pharmaceutical Sciences

2-5-1 Shikatacho, Kitaku, Okayama, Japan 700-8558

Email: [yasuhiro.kotani@cc.okayama-u.ac.jp](mailto:yasuhiro.kotani@cc.okayama-u.ac.jp)

**Online resource 1. Preoperative variables associated with early outcomes**

| **Author, year** | **N** | **Endpoint** | **Variables** | | | | | | | | | |
| --- | --- | --- | --- | --- | --- | --- | --- | --- | --- | --- | --- | --- |
|  |  |  | **Weight** | **Truncal valve regurgitation** | **IAA** | **Shock** | **Prematurity** | **Coronary anomaly** | | | **DiGeorge syndrome** | |
| Mastropietro, 2019 | 216 | MACE | No | No | – | Yes | No | No | | | No | |
| Herrmann, 2020 | 103 | Early mortality | No | – | Yes | – | – | No | | | – | |
| Naimo, 2020 | 255 | Early mortality | Yes (<2.5 kg) | No | Yes | – | – | – | – | | |  |
| Guariento, 2022 | 204 | Early mortality | No | Yes | No | – | No | No | | No | |  |
| Goyal, 2023 | 647 | Hospital mortality | Yes (<2.5 kg) | Truncal valve surgery | No | – | – | No | | No | |  |

*IAA*, interrupted aortic arch; *MACE*, major cardiac adverse events.
