# Supplementary material for: Management Strategies for Truncus Arteriosus: A Comparative Analysis of Staged vs. Primary Repair
Source: Pediatr Cardiol. 2025 Jan 30;47(2):514–22. doi: 10.1007/s00246-025-03790-z (PMC12855218; doi:10.1007/s00246-025-03790-z)
Supplement: Supplementary file 2 — Supplementary file2 (DOCX 57 KB) [file 246_2025_3790_MOESM2_ESM.docx]

**Management strategies for truncus arteriosus: a comparative analysis of staged vs. primary repair**

*Pediatric Cardiology*

Yasuyuki Kobayashi, Shunji Sano, Yuto Narumiya, Ayari Kimura, Etsuji Suzuki, Shingo Kasahara, Yasuhiro Kotani*

*Corresponding Author:

Yasuhiro Kotani, MD, PhD

Department of Cardiovascular Surgery

Okayama University Graduate School of Medicine, Dentistry, and Pharmaceutical Sciences

2-5-1 Shikatacho, Kitaku, Okayama, Japan 700-8558

Email: [yasuhiro.kotani@cc.okayama-u.ac.jp](mailto:yasuhiro.kotani@cc.okayama-u.ac.jp)

**Online Resource 2. Summary of mortality**

| **Case** | **Group** | **Timing of mortality** | **Risk** | **Course** |  |  |
| --- | --- | --- | --- | --- | --- | --- |
| 1 | Primary | Hospital | IAA, preoperative shock | Patient (3.2 kg, day 13), who was transferred for ductal shock, died of subsequent multiorgan failure after repair on POD 8. | |  |
| 2 | Primary | Hospital | Preoperative shock | Patient (3.7 kg, day 35), who was transferred for cardiogenic shock with pulmonary hypertension crisis, died of multiorgan failure after repair on POD 33. | |  |
| 3 | Primary | Hospital | Weight ≤2.5 kg | Patient (2.4 kg, day 7) had desaturation due to pulmonary stenosis at the distal anastomosis site, requiring emergent extracorporeal membrane oxygenation, which was later discontinued due to hypoxic encephalopathy following family consultation on POD 48. | | |
| 4 | Primary | Hospital | Weight ≤2.5 kg | Patient (1.9 kg, day 13, term delivery) died of massive subarachnoid hemorrhage after repair on POD 8. | | |
| 5 | Primary | Late | ≥Moderate truncal valve regurgitation | Patient (2.6 kg, day 21), who underwent truncal valve repair for moderate truncal valve regurgitation, had persistent truncal valve regurgitation and chronic biventricular failure and subsequently experienced cardiac arrest at home at the age of 4 years. | | |
| 6 | Staged | Late | Weight ≤2.5 kg | Patient (1.8 kg, day 19, term delivery) with Cornelia de Lange syndrome, who did not have any complications after bPAB followed by repair (5.8 kg, day 460), died of pneumonia at the age of 4 years. | | |

*IAA*, interruption of the aorta; *POD*, postoperative day.
